# Promoting Healthier Meal Selection and Intake Among Children in Restaurants: Protocol for a Cluster-Randomized Trial

**DOI:** 10.2196/73618

**Published:** 2025-10-10

**Authors:** Stephanie Anzman-Frasca, Sara Tauriello, Leonard Epstein, Mackenzie J Ferrante, April Gampp, Juliana Goldsmith, Jess Haines, Lucia A Leone, Rocco Paluch

**Affiliations:** 1 Department of Pediatrics Jacobs School of Medicine and Biomedical Sciences University at Buffalo, State University of New York Buffalo, NY United States; 2 Center for Ingestive Behavior Research University at Buffalo, State University of New York Buffalo, NY United States; 3 Department of Nutritional Sciences School of Environmental and Biological Sciences Rutgers, The State University of New Jersey New Brunswick, NJ United States; 4 Independent Health Foundation Buffalo, NY United States; 5 Department of Family Relations and Applied Nutrition University of Guelph Guelph, ON Canada; 6 Department of Community Health and Health Behavior School of Public Health and Health Professions University at Buffalo Buffalo, NY United States

**Keywords:** restaurant, choice architecture, repeated exposure, menu, child, diet, health

## Abstract

**Background:**

US children’s diets are high in calories and are of poor nutritional quality, and a likely contributing factor is the consumption of food from restaurants. While children readily accept the sweet and salty foods that characterize restaurant children’s menus, research shows that their taste preferences are malleable, and regular exposure to healthier foods can promote their acceptance.

**Objective:**

We describe a cluster-randomized controlled trial testing the effects of behavioral intervention strategies (choice architecture and repeated exposure) on ordering and dietary intake among children in restaurants and present baseline demographic data for the study cohort.

**Methods:**

Six locations of a regional quick-service restaurant chain were randomized to the intervention or control group in pairs based on income in surrounding census tracts. Families with children aged 4 to 8 years were recruited and asked to complete 8 visits to the study restaurant, including a baseline assessment completed at the time of enrollment, followed by 6 visits during a designated 2-month exposure period and a final posttest assessment. Intervention content provided to intervention group families after baseline assessments includes placemats promoting 2 healthier kids’ meals and the opportunity to redeem their kids’ meal “cone token” for a toy instead of a dessert (choice architecture strategies). In addition, participating families receive frequent diner cards, which can be used to earn a free kids’ meal after purchasing a promoted kids’ meal 6 times (repeated exposure strategy). Families in control restaurants receive generic versions of these materials (eg, frequent diner cards that can be redeemed for a free kids’ meal after purchasing any 6 kids’ meals). The primary outcome is the meal ordered for the child at a posttest restaurant visit following the exposure period (ie, whether or not a promoted meal was ordered). Additional order data will include calories, saturated fat, sodium, and sugar content of children’s orders at posttest. Other outcomes include children’s in-restaurant and daily consumption of calories, saturated fat, sodium, and sugar.

**Results:**

This study was funded in 2019, with preregistration completed in 2020, data collection occurring from June 2021 to November 2024, and data processing, analysis, and primary outcome manuscript preparation in 2025-2026. A total of 236 families provided baseline data on children’s orders and comprise the study cohort; 234 of these families provided demographic data (n=184, 78.3% female parents; n=133, 56.8% female children; child mean age 6.5, SD 1.3 years).

**Conclusions:**

Given that restaurants are normative eating contexts for many children, this intervention has the potential to impact children’s dietary intake and health. If found to be successful, future directions could include scaling the current intervention approach and conducting further effectiveness, implementation, and dissemination research to understand its applicability and impact across different types of restaurants and sociodemographic contexts.

**Trial Registration:**

ClinicalTrials.gov NCT04334525; https://clinicaltrials.gov/study/NCT04334525

**International Registered Report Identifier (IRRID):**

DERR1-10.2196/73618

## Introduction

US children’s diets are generally of poor nutritional quality [1]. A likely contributing factor is frequent consumption of food from restaurants, where meals tend to be higher in energy content and lower in nutritional quality than those prepared at home [2]. While young children readily accept the sweet and salty foods that tend to characterize typical restaurant meals [3-5], research shows that their taste preferences are malleable, and regular exposure to healthier foods can promote acceptance of these foods beginning early in life [6,7]. In this paper, we describe a 2-arm cluster-randomized controlled trial testing the effectiveness of an intervention using evidence-based approaches from the behavioral sciences to promote healthier eating among children in quick-service (ie, fast food) restaurants.

Studies have shown that the minority of children’s meals offered at quick-service and full-service restaurant chains meet nutritional recommendations [3-5]. In turn, consumption of restaurant food has been linked with increased daily energy intake and poor diet quality among children, including higher saturated fat and sugar intake [8]. Furthermore, restaurants have become normative eating contexts for many families, with more than one-third of children eating fast food on a given day [9]. Given the frequency at which children eat from restaurants and typical consumption patterns while there, targeting children’s food selection in restaurants has the potential to improve diet quality and shape healthy eating habits.

A nationally representative study showed that more than half of children would accept fruits or vegetables with children’s meals, but an even higher percentage of children reported being likely to order meals that came with French fries [10], suggesting that the promotion of healthier options may be most successful when these options are not in direct competition with less-healthy, familiar offerings. In other words, if children have a choice between a fruit or vegetable side versus French fries, many may choose French fries, but if fruits and vegetables are made the default option, many children would be satisfied with these options.

Choice architecture approaches that increase the prominence of healthier choices can facilitate the selection of these options. Choice architecture approaches modify the environment in which decisions are made and have demonstrated success in nudging consumers toward a particular option without overt health messages or the removal of choice. For example, making healthier side dishes the default offering with children’s meals has been linked with healthier meal selection in quick-service [11] and full-service restaurants [12,13]. The power of defaults is also supported by extensive behavioral economics research in other domains outside the realm of food choice (eg, [14,15]). There is also evidence that modifying the prominence of target items by using engaging names [16] or serving target items before the main meal [17] can increase children’s consumption of those foods, with this research conducted in a childcare center and in a university campus-based, full-service restaurant, respectively. Although these strategies show promise, there is limited research examining interventions intended to nudge children toward healthier meals in restaurants, including in quick-service restaurant chains that are frequented regularly by families. Our team conducted a pilot study to begin filling this gap. In this study, families were recruited from a single location of a regional quick-service restaurant chain and randomly assigned to return during a 2-week intervention or control period. During the intervention period, families received placemats that featured 2 healthier kids’ meals, positioning these options as special (“Kids’ Meals of the Day”), prominent (listing only the featured meals on the placemats), fun (using names like The Nutty Monkey meal), and automatic (bundling healthy sides and beverages with featured main dishes). Children who received these placemats ordered a greater number of healthier foods than controls, and children who ordered promoted healthier main dishes consumed less saturated fat than those who did not [18]. The relatively small effect sizes observed, along with a trend where controls rated their meals as more palatable compared to intervention group children, highlighted opportunities to bolster this pilot study. This trial was designed to build upon this work through an in-restaurant intervention that (1) takes place along a longer timeline, (2) promotes healthier meals previously rated by children as palatable, and (3) combines the choice architecture strategies used in the pilot study with repeated exposure.

A robust evidence base supports simple repeated exposure to tastes of target foods as one of the most effective ways to promote healthier food preferences and intake among children [6,7], with recent research suggesting that 6 exposures or less can be enough to increase acceptance of target foods [19-22]. To our knowledge, this approach has not been studied systematically in restaurants; however, it fits well with the goal of promoting healthier eating in restaurants as taste is a leading factor influencing the meals children select in these settings [23]. For this trial, our intervention combined choice architecture—that is, an evidence-based behavioral economics approach designed to nudge participants’ attention toward 2 healthier children’s meals—with a repeated exposure intervention in the form of a frequent diner card designed to incentivize continued ordering of the promoted healthy meals and build preferences for these meals through these exposures over time.

For this cluster-randomized trial, our team is working with the same restaurant chain as in our pilot study, this time randomizing all 6 of its locations to intervention or control. The same kids’ meals are available in the intervention and control restaurants, but in intervention restaurants, choice architecture and repeated exposure strategies are used to promote 2 healthier children’s meals. Order and intake data of participating children aged 4 to 8 years are collected in the restaurant at baseline and again at a posttest visit following a 2-month exposure period. During the exposure period, it is recommended that families visit the restaurant at least 6 times, and weekly internet-based surveys are administered to parents. The recommended 6 restaurant visits during the exposure period correspond to the frequency that participants typically dine out, as only families who dine out at least 2-3 times per month were eligible for the study. The primary outcome is whether or not the child orders one of the promoted healthier kids’ meals at the posttest assessment following exposure to the intervention strategies. It is hypothesized that children in intervention restaurants will be more likely to order the healthier kids’ meals, which were promoted in their restaurant location, and will demonstrate healthier orders (lower calories, saturated fat, sugar, and sodium) and intake (lower calories, saturated fat, sugar, and sodium) in the restaurant.

Taken together, research to date suggests that repeated exposure and choice architecture have the potential to make healthier options more appealing and easier to choose. Given how frequently children eat food from restaurants, the typical unhealthy consumption patterns while there, and the evidence supporting the potential of repeated exposure to impact eating behaviors broadly, this intervention has the potential for meaningful impacts on children’s diets and health.

## Methods

### Overview

In this 2-arm cluster-randomized controlled trial, all 6 locations of Anderson’s Frozen Custard, a quick-service restaurant chain in the Western New York region of the United States, were paired based on poverty levels in the surrounding census tracts before participant enrollment (ie, 0% and 4%, 10.2% and 10.3%, and 29% and 38.1% living below the federal poverty level), and a location from each pair was randomized to either the intervention or control group by a study statistician (RP). The locations were paired before randomization to avoid an imbalance in socioeconomic status across the study groups. While participants could see the specific intervention materials described herein after their baseline assessments, they were not told that they were in an intervention versus control group specifically; it was not possible to mask data collectors from study group assignment.

Data collection was planned to take place over 3 years, with each pair of restaurants assigned a priori to 1 of 3 cohorts (see Adaptations Due to COVID-19 section for details on slight modifications made due to the emergence of the COVID-19 pandemic at the start of the planned study timeline). Families with children aged 4 to 8 years were recruited at each location, with a focus on the age range expected to order from the children’s menu. Eligible, enrolled families were asked to complete 8 visits to the restaurant location where they were recruited. Visits include a baseline visit (with data collected following recruitment), followed by 6 visits within a specified 2-month exposure period, and finally, a posttest visit, with in-person assessments at the initial and posttest time points. Data collection methods are consistent with our pilot study [18]. Detailed procedures are described herein.

### Recruitment

During the baseline period, study staff were present in the restaurant during designated days and times (generally from Wednesday to Saturday between 4:30 PM and 8 PM), and families with children who entered participating restaurant locations during those times were screened for interest and eligibility. One adult and one child were recruited from each interested, eligible family. Inclusion criteria were: participating child aged 4 to 8 years, with the parent or guardian reporting that the child normally eats food from restaurants at least 2-3 times per month. The participating adult also had to be 18 years of age or older, and the child’s parent or legal guardian, and both the parent and child had to be English speaking and planning to order a meal for the child in the restaurant that day. Parent-child dyads were excluded if the above criteria were not met, if the child had allergies that would preclude safe participation, or if the child participated in a pilot study involving taste tests of the study’s featured healthier kids’ meals in 2019 [24]. If multiple children within a family met eligibility criteria, only a single child was enrolled, with the study team recommending the child with the most recent birthday. Baseline data collection procedures described herein were administered after the family finished their meals on the recruitment day, with intervention materials to be provided thereafter.

### Ethical Considerations

All study procedures were approved by the University at Buffalo Institutional Review Board (STUDY00003356) and registered at clinicaltrials.gov (NCT04334525) before participant enrollment. Participants could receive up to US $265 for successful participation or up to US $300 if they were selected to and decided to participate in an additional, optional observational substudy [25]. All adult participants provided informed consent and permission for their children’s participation, and children provided assent before commencement of study procedures. Given the minimal risk of the study, there are no stopping criteria. Participants are informed that their participation is voluntary and that they can discontinue their participation at any time. After each data collection day, a daily debrief is sent to the principal investigator to allow monitoring for any adverse events. Data collected to assess study aims are stored separately from identifiers. Identifiable information (eg, names and contact information) is stored securely and separately and will be destroyed within 10 years of the study’s conclusion.

### Sample Size Calculation

The primary outcome for this trial is whether or not the child ordered one of the promoted healthier children’s meals at posttest. We estimated that about 27.8% of participants exposed to intervention content would order promoted healthier meals versus 6.7% of the control group [18]. Using these values, an alpha of .05, power of 80%, 6 clusters, and an intraclass correlation of .01 based on previous restaurant research [12], it was estimated that 124 families would be needed to detect intervention effects on meal selection. Considering return rates from previous restaurant research [26], as well as the plan to explore additional outcomes, we increased this estimate by 35% in estimating the sample size for primary outcome analysis, resulting in a target sample size of at least 168 families (84 per group), with our enrolled study cohort exceeding this target.

### Intervention

#### Overview

After baseline assessments, families are asked to return and dine at the restaurant location where they were recruited 6 times over a 2-month period during which intervention components are in place. Those recruited in restaurant locations that had been randomized to the intervention group experience an in-restaurant intervention that combines choice architecture and repeated exposure strategies to promote 2 healthier kids’ meals.

#### Choice Architecture

**Figure 1:**
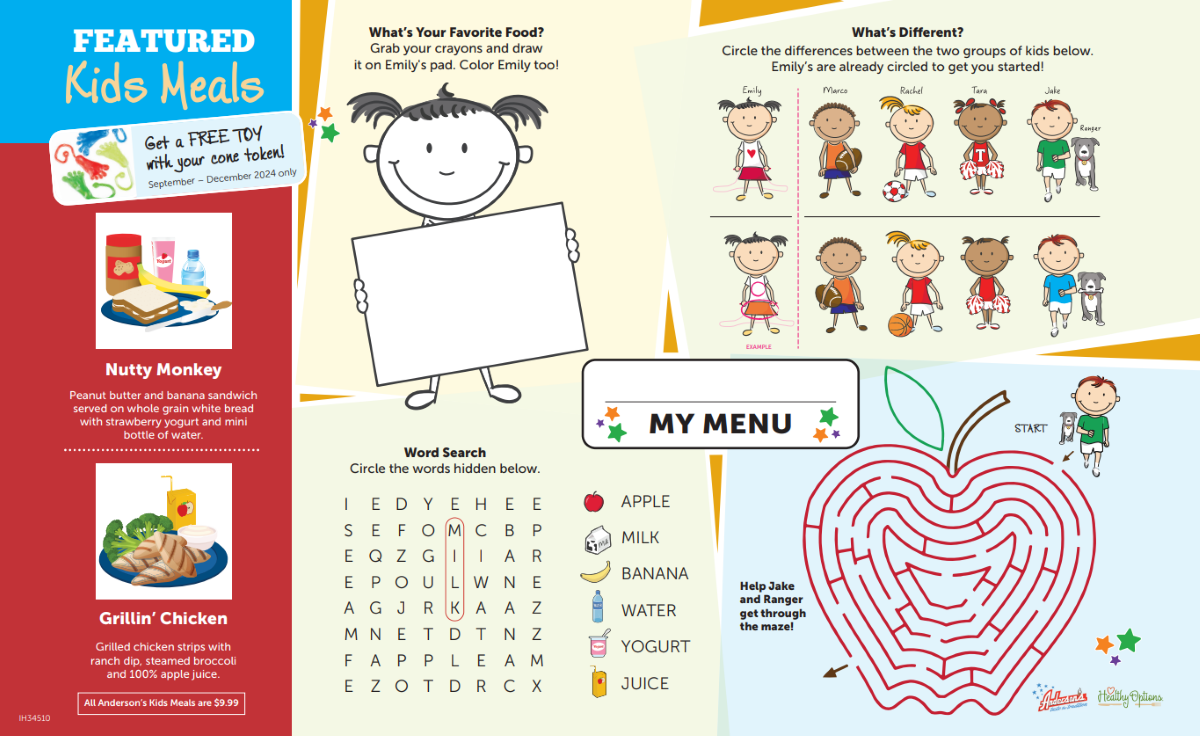
Sample placemat for the intervention group.

Families in intervention restaurants receive crayons and placemats promoting 2 healthier kids’ meals during the exposure period (see Figure 1). Placemats are mailed to enrolled families after their baseline assessments and are also available to all families dining in the intervention locations, whether or not they are participating in the study, throughout the exposure and post periods. The placemats are available to take from a child-friendly display; restaurant staff are also trained to include a placemat and a set of crayons with every kids’ meal order. Promoted meals were selected based on formative research conducted before the main study. In this formative work, 10 possible main and side dishes were taste-tested by 37 children aged 4 to 8 years across two locations of the study restaurant [24]. Children rated their liking and rank-ordered preference for each food option. Highly-rated meal components included grilled chicken strips with ranch dip, a peanut butter and banana sandwich, steamed broccoli, and strawberry yogurt. We bundled these preferred items into 2 featured kids’ meals, along with preferred beverages (100% apple juice and water), with each bundled meal meeting nutritional criteria corresponding to the National Restaurant Association’s Kids’ Live Well program and the Dietary Guidelines for Americans [27,28].

As in our pilot study [18], intervention placemats position the 2 promoted meals as special (“Kids’ Meals of the Day”), prominent (ie, featuring only them on the placemats), fun (ie, with fun names like The Nutty Monkey and Grillin’ Chicken), and automatic (ie, bundling healthier sides and beverages with featured main dishes). Placemats also aim to shift the default nature of desserts by promoting the opportunity for children to redeem the “cone token” that comes with all kids’ meals at this restaurant for a small toy (eg, dinosaur building block set or squishy toy animal) instead of a dessert. Final toy options were selected after brief focus groups conducted with a separate, small group of children in the same age range. Like the placemats, the toy promotion is available to all customers in the restaurant during the exposure and posttest periods. Signage that reinforces the messages from the placemats (ie, promoting the featured meals and toys) is also displayed in the intervention restaurant locations during the exposure and posttest periods, including a large sign posted next to the kids’ menu board above the cash register and smaller signs displayed throughout the restaurant. The placemats and signs used were the same throughout each year’s exposure and posttest period, with minimal adjustments from year to year to reflect minor details such as the current year and current kids’ meal prices.

#### Repeated Exposure

**Figure 2:**
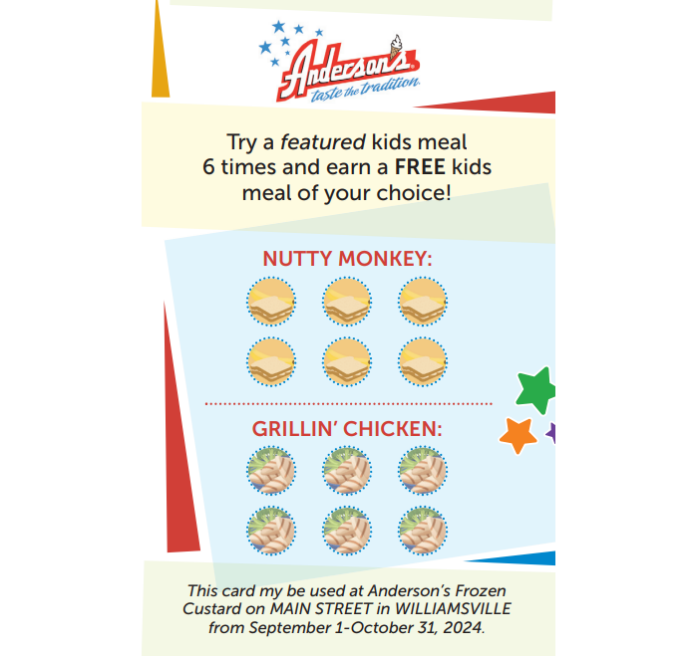
Sample frequent diner card for the intervention group.

After baseline data collection, participating families also receive frequent diner cards for use during the study’s exposure period. In intervention restaurants, participating families can earn a free kids’ meal at posttest if the child orders one of the promoted healthier meals 6 times during the designated 2-month exposure period. These cards are provided electronically, with a link to an image (see Figure 2) sent to each participating family, which features a code that can be scanned in the restaurant. When providing the cards to families, study staff clarify which 2 specific meals can be used to earn a scan of the digital frequent diner card, and that either one of these meals may be purchased. When a promoted meal is purchased, restaurant staff scan the card, and the child gets to choose a sticker. After using the card for an eligible meal 6 times, a free kids’ meal automatically populates in the participant’s account. The frequent diner card can be used once per visit and only by the participating child. Unlike the placemats, signage, and toys, this component of the study is available to study participants only.

#### Control Group

Control-group families receive crayons and generic placemats featuring the restaurant’s full kids’ menu, with these materials and corresponding signage displayed in control restaurants during the exposure and posttest periods. The components of the 2 meals that are promoted as part of the study are available in control restaurants as part of the kids’ menu, but are not bundled together or promoted in any way. In contrast to intervention group materials, the generic placemats and signs do not advertise the promoted meals over and above any other kids’ meals, nor do they note the opportunity to redeem the kids’ meal token for a toy instead of a dessert. Control families also receive a frequent diner card, which can be scanned when purchasing any kids’ meal during the exposure period. Children in this group earn a free kids’ meal at posttest if any 6 kids’ meals are purchased during the 2-month exposure period. There was no toy promotion in the control group. Both groups receive study payments for study activities that they complete, including attending the posttest session, whether or not the free kids’ meal is earned.

### Measures

#### Overview

At both the baseline and posttest assessments, study staff collect order and intake data in person, after the family has finished their meal. Table 1 shows detailed study outcomes by time point and measure. The final paragraph of the measures section describes slight modifications to procedures and measures that were necessary during the first (2021) cohort only, due to the COVID-19 pandemic.

**Table 1:** Planned study outcomes by time point and measure.

Study outcomes		Baseline	Exposure period	Posttest		
**Children’s restaurant meal orders**						
	Items ordered for children in the study sample^a^	P^b^	P	P^c^		
	Total calories, saturated fat, sodium, and sugar in child meal orders^a^	P, N^d^	P, N	P, N		
	Children’s meal orders in study restaurants overall during study	S^e^	S	S		
**Children’s intake: in the restaurant and total daily**						
	Total calories, saturated fat, sodium, and sugar consumed in a restaurant^a^	W^f^, N	—^g^	W, N		
	Total daily calories, saturated fat, sodium, and sugar consumed.^a^	—	—	P (ASA24^h^)		
**Other variables of interest**						
	Demographics^a^	P	—	—		
	Parent restaurant meal orders^a^	P, N	P, N	P, N		
	Process measures (parent and child perspectives, tracking intervention materials, and restaurant staff perspectives) ^a^	P, C^i^	P, I^j^, O^k^	P, C, O		
	Observations of parent-child interactions in the restaurant^a^	—	O^l^	—		

^a^Depicts individual-level measures.

^b^P: parent report.

^c^Primary outcome.

^d^N: nutrition information from the restaurant.

^e^S: sales data.

^f^W: weighed plate waste.

^g^Not collected at this time point.

^h^ASA24: Automated Self-administered 24-hour Dietary Assessment Tool.

^i^C: child report.

^j^I: restaurant staff interview.

^k^O: observation.

^l^Observed in a random subsample once during the 2-month exposure period.

#### Order Content and Liking

To assess the impact of the intervention on the meal ordered for the child (primary outcome), study staff first ask the participating parent to identify all items ordered for and eaten by the participating child in the restaurant that day and record the specific main dish(es), side dish(es), beverage(s), and dessert(s) ordered for and eaten by the child, noting whether each item was shared, will be taken to go, or was finished in the restaurant, as well as whether the child used the token that comes with all kids’ meals to select a dessert, toy, or neither. Total calories, saturated fat, sugar, and sodium ordered for the child are calculated using nutrition information from the restaurant. Parents also report what they ordered for themselves as part of the self-administered survey described below. In addition to the main, in-person assessments of orders, links to brief internet-based surveys are sent to participating parents electronically (typically via text message, with email communication also available when preferred) each week of the exposure period. Surveys include questions about the family’s visit(s) to the study restaurant during the past week, including the parent’s and the child’s meal selections and liking of the meals. Calories, saturated fat, sugar, and sodium ordered are calculated from this information as described above.

#### Dietary Intake in the Restaurant

To assess the intervention effect on children’s intake during their meal, plate waste methodology is used during the in-person visits at baseline and posttest [18,29]. Participating families are instructed to avoid throwing away any leftover food or trash until our team has collected information from them after their meal. After asking the parent which items were ordered for and consumed by the participating child, study staff collect, package, and label all corresponding uneaten food and containers from the child’s meal. Items are postweighed in the laboratory following data collection, with pre-to-post weight differences indicating grams consumed. For any items the child wishes to take home with them, the postweight is instead taken in the restaurant. Preweights are estimated by purchasing 3 replicates of each food/beverage item ordered for children during the study and averaging their weights to arrive at a standard preweight. Grams consumed are converted to percentages consumed using total grams from preweights, and these percentages are multiplied by the total calories, saturated fat, sodium, and sugar found in the full item to calculate calories and nutrients consumed.

#### Total Daily Dietary Intake

Following the posttest assessment, parents report children’s daily dietary intake using the Automated Self-administered 24-hour Dietary Assessment Tool (ASA24). Parents are asked to report their children’s dietary intake on the day immediately after the posttest restaurant visit. A previous validation study of the ASA24 with 40 parents of children aged 2 to 5 years showed that parent report of child intake matched that of observed dietary intake with nearly 80% accuracy [30]. These data will be used to examine potential intervention effects on children’s daily intake of energy, sugar, saturated fat, and sodium, to shed light on potential broader impacts of the intervention, as well as the potential for any compensation.

#### Demographics and Restaurant Experiences

After the parent is interviewed about the child’s order at baseline and posttest, parents are provided with an electronic tablet to self-report on other aspects of the child’s restaurant meal that day, and at baseline only, demographics within a brief self-administered internet-based survey.

##### Demographics

At baseline, parents report their age, sex, race, ethnicity, education level, and income, and their child’s age, sex, race, ethnicity, height, and weight.

##### Restaurant Experiences

When completing the internet-based survey on site at both the baseline and posttest assessments, parents also indicate who selected the child’s meal, the reason for the meal choice, how often the child eats at restaurants, and how often the child eats at the study restaurant, using questions adapted from our previous research [10]. At posttest, parents also report on their child’s visits to the study restaurant since the last study contact and their perspectives on the study materials.

#### Child Perspectives

While parents are self-administering surveys at baseline and posttest, a study staff member asks the child: whether they knew what they would be having before arriving at the restaurant, whether they had been to this restaurant before, whether this was the meal they typically ordered, who selected the meal, and how they liked their meal [10]. In addition, at posttest, children answer questions about study materials (see Multimedia Appendix 1). Visual aids are used to assist children with answering the questions (eg, smiley face scale for indicating how much they liked their meal). Children’s responses are entered into an electronic tablet by the study staff member asking the questions.

#### Other Measures

In addition to the main measures collected at baseline, the assessments during the exposure period, and posttest, several other sources of data are collected as part of the study. These include sales data, intervention implementation tracking, restaurant staff interviews, and parent-child observations, each of which is described briefly below. Components that are considered to be process measures include the implementation tracking and restaurant staff interviews, as well as parents’ and children’s perspectives on the intervention collected via the aforementioned surveys (see Table 1).

#### Sales Data

Aggregated sales data from each participating restaurant location are collected to monitor the sales of healthier and all kids’ meals across all customers during the baseline, exposure, and posttest periods within each of the cohorts and parallel time points 1 and 2 years before. These data will allow an examination of overarching trends in the purchases of the healthier kids’ meals in intervention versus control restaurants over the course of the study, which can provide insights into the impacts of the choice architecture intervention components on a larger scale, with less direct researcher involvement.

#### Intervention Implementation Tracking

During each week that intervention materials are on display at study locations, a study coordinator visits each restaurant location and records whether study materials are displayed as intended. Quantities of study materials used are also recorded (eg, number of placemats distributed by restaurant locations; number of toys requested at the intervention locations). This information, as well as data on the use of the frequent diner cards, will be aggregated and summarized at the end of the study.

#### Restaurant Staff Interviews

During each study year, at least 1 month into the exposure period, restaurant staff are invited to participate in semistructured interviews to share their perspectives on the study. Question development was guided by the Consolidated Framework for Implementation Research (CFIR) [31]; questions include how familiar the respondent is with the study objectives, how easy or hard it is to implement different aspects of the study interventions, and whether these are a good fit for the restaurant. The restaurant owner, managers, and other employees are invited to participate in interviews, with a slightly different set of interview questions depending on their role (see Multimedia Appendices 2 and 3). In each study year, recruitment for the interviews continues into early November or until our sample size quota for that year is satisfied. We planned to complete up to n=12 interviews per year, aiming for representation of each restaurant location, as well as representation across the different roles (eg, managers and other employees) each year.

#### Observations of Family Interactions

Finally, parent-child interactions are observed as an ancillary measure to provide descriptive information to contextualize this study’s results and also generate hypotheses for future research, given the current lack of observational data on parent-child eating occasions in restaurants. These observations allow examination of parent-child interactions in this setting generally (eg, parenting behaviors), as well as families’ engagement with study materials.

Parent-child interactions during meals are observed during the exposure period. The original goal was to conduct observations on about 10% of the total sample. We randomly selected one year (2023/Cohort 3) during which these optional observations would take place. Interested families are recruited into the optional observation component of the study on a first-come first-served basis. Participating families are scheduled for an observation during one of their regular visits to the study restaurant during the exposure period. Unobtrusive observations take place during the first 5 minutes after families are seated at their table and the first 5 minutes they are eating, following procedures adapted from previous research [32,33]. The 5-minute intervals are a standard time frame used in the behavioral coding system from which the study procedures were adapted. Trained, reliable observers collect data on key variables of interest [25].

#### Adaptations Due to COVID-19

The study procedures and measures described herein correspond to procedures used between 2022 and 2024. During 2021, the first year of data collection, modifications were necessary due to the COVID-19 pandemic, impacting 11% of the study cohort (n=26). The main change was that the research team collected data remotely, rather than being in the restaurant with participants, primarily affecting recruitment methods and the collection of baseline and posttest data. The restaurant was open, and customers could visit as usual. Given that it was not appropriate for us to approach customers in person during this time, we relied on passive recruitment methods, posting opportunities to sign up for the study throughout the restaurant, which included avenues for learning more about the study and for contacting our team. Our team was working off-site Monday through Sunday evenings, fielding inquiries, screening and enrolling individuals into the study, and administering baseline assessments using a text messaging system and internet-based surveys. The questions about children’s meal orders were the same as those of the other cohorts, but were administered via internet-based survey rather than starting with an interview. Weighed plate waste measurements were not possible under these circumstances, so we used food photography methods to approximate child intake at posttest. Details of those procedures and a subsequent study of their validity will be presented separately. Children were not interviewed during this first cohort. All other study components were as originally intended and thus consistent across cohorts (eg, provision and display of intervention materials, administration and completion of internet-based surveys during the exposure period). Given these changes to the first cohort, we added a fourth cohort, so we would still be able to conduct in-person data collection with each restaurant location and reach sample size goals.

#### Statistical Analyses

To examine preregistered aims, mixed models will be used to examine the effects of the intervention on meals ordered for children and children’s intake. Key questions will be whether children’s restaurant meal orders differ between the intervention and control group at the posttest assessment, with specific outcomes of interest including whether or not a promoted healthier meal was ordered for the child, whether a dessert was ordered, and calories, saturated fat, sodium, and sugar ordered. We will also examine group differences in calories, saturated fat, sodium, and sugar consumed by the child at posttest and changes in the order and intake outcomes over the course of the study by group.

Before implementing the mixed models, we will conduct an assessment of the randomized unit of our cluster-randomized trial, restaurant location, examining the variance component estimates in each model. When cluster variance is either estimated as effectively 0 or negative, we will report the cluster intraclass correlation and omit the random effect of restaurant location from our model. With only 6 unique locations being randomized in our trial, small cluster size adjustments will be applied to any models with the random effect of restaurant location retained, to improve type I error rates [34]. More specific details about the analyses that will be used to address each study aim follow.

For dichotomous outcomes (eg, whether or not one of the promoted healthier meals was ordered), we plan to use logistic regression with generalized linear mixed models, which have the flexibility to handle dichotomous outcome distributions. Models will include fixed effects of group, time point, and the group*time point interaction, with an adjustment for baseline status on the outcome variable and a random effect of participant (ie, repeated time points within persons). Restaurant location will be included as a random effect pending tests of clustering. Logistic generalized linear mixed models will use a binomial distribution and a logit link function. The percentage of participants in each study group who ordered the healthy promoted meal items will be described over time, with a corresponding figure illustrating the percentage ordering the healthy meal at each time point. Analyses predicting ordering of healthier promoted meals will be conducted once with the promoted meals strictly defined: that is, must include a promoted main dish, side dish, and beverage, and again with two less restrictive definitions: (1) considering the meal to be healthier if it included at least the promoted main dish and (2) considering the meal to be healthier if it included at least one of the promoted items. We will use the same analytic approach for the analysis of whether or not a dessert was ordered for the child in the restaurant.

For continuous outcomes (eg, total calories, saturated fat, sodium, and sugar ordered for the child in the restaurant), we will use mixed models to examine intervention effects across baseline, all exposures, and posttest. Models will include fixed effects of group, time point, the group*time point interaction, and baseline outcome, with a random effect of individual participant. Restaurant location will also be included as a random effect pending tests of clustering. Parallel models will be conducted to examine intervention effects on children’s consumption, including calories, saturated fat, sodium, and sugar consumed during the restaurant meal. For the dietary intake models, only baseline and posttest data points will be used, given that intake data were not measured during the exposure period.

We will also examine: (1) associations between selection of healthy promoted meal items and nutritional characteristics of children’s meal orders and intake, (2) potential covariates and effect moderators (eg, child sex, age, weight status, and who selected the child’s meal order), (3) intervention effects on children’s 24-hour intake, and (4) intervention impacts on ordering of healthier items in aggregate sales data. Data from implementation tracking will be summarized. Restaurant staff interviews are recorded, transcribed, and checked for accuracy. A codebook will be developed using a combination of inductive and deductive approaches guided by CFIR [31] in collaboration with the study principal investigator (SAF) and a co-investigator with expertise in qualitative methods (LAL). The transcripts will be independently coded by 2 trained coders using Atlas.ti (Lumivero) qualitative analysis software. Codes will be reviewed and summarized into themes represented by illustrative quotes. Analysis of data from the parent-child observation substudy, which concluded before the main study, has been published previously [25].

We will conduct sensitivity analyses to examine the robustness of the primary results, including conducting our primary data analyses with and without the first cohort included, to see whether the modified mode of data collection during the COVID-19 pandemic impacts study results. The main analysis plan is to include all participants who provided baseline child order data, but supplemental analyses will be presented as appropriate if sensitivity analyses demonstrate impacts on study results.

## Results

**Figure 3:**
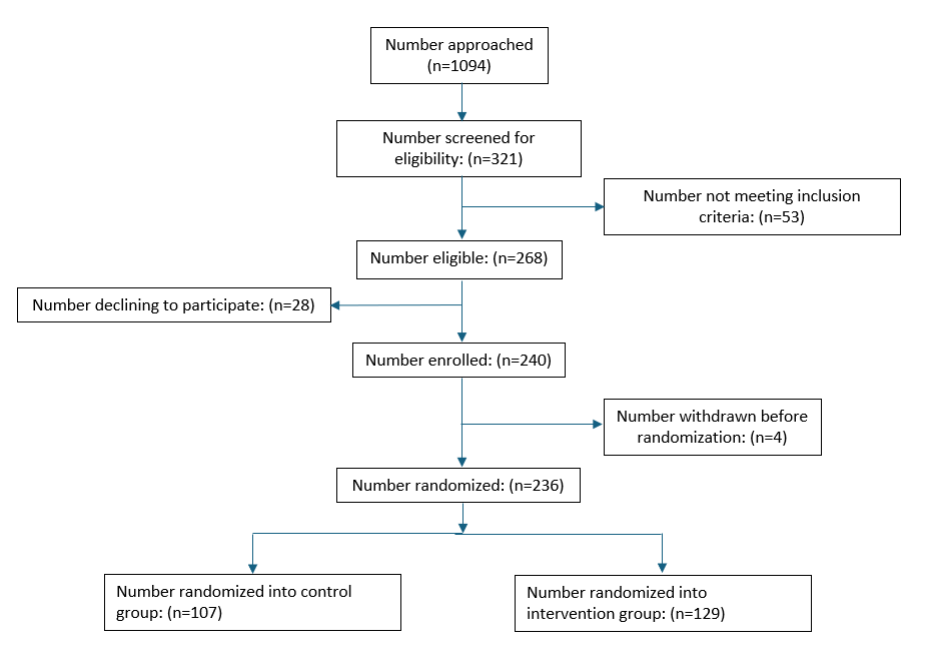
CONSORT (Consolidated Standards of Reporting Trials) study flow diagram through randomization.

This study was funded in 2019, with preregistration completed in 2020, data collection occurring from June 2021 to November 2024, and data processing, analysis, and primary outcome paper preparation in 2025-2026. Participants in restaurant locations A and B were recruited in the Summer of 2021, those in C and D were recruited in the Summer of 2022, and those in E and F were recruited in the Summer of 2023. Modifications to procedures were needed in 2021 due to the COVID-19 pandemic, as described above. Given these modifications, as well as the fact that fewer families than anticipated participated during this initial, pandemic-impacted year, we recruited an additional, fourth group of participants: in 2024, we recruited (different) families from restaurant locations A and B, allowing us to reach our target sample size and to collect data from families in each restaurant location using the originally planned study procedures. In total, 1094 families were approached, 268 were eligible, 240 enrolled in the study, and 236 provided baseline data on children’s meal orders and are considered the study cohort (see Figure 3 for the CONSORT [Consolidated Standards of Reporting Trials] diagram). Among the 234 study cohort families providing demographic data, 184 (78.3%) of parents were female, 133 (56.8%) of children were female, and the average child age was 6.5 years (SD 1.3 y). Most children were white (196; 83.8%); 24 (10.3%) were multiracial, 11 (4.7%) Hispanic or Latino, 10 (4.3%) Black, and 1 (0.4%) Asian. Complete baseline demographic characteristics of the study cohort are shown in Table 2.

**Table 2:** Demographic characteristics of the study cohort at baseline (N=234).

Characteristic		Results^a^				
**Parent sex, n (%)**						
	Female	184 (78.3)				
	Male	50 (21.3)				
Parent age (years), mean (SD)		39.4 (7.5)				
**Parent race and ethnicity, n (%)**						
	White	212 (90.6)				
	Black	12 (5.1)				
	Asian	3 (1.3)				
	Multiracial	5 (2.1)				
	Hispanic or Latino	8 (3.4)				
**Parent education, n (%)**						
	<High school graduate or equivalent	16 (6.8)				
	Some college or associate’s degree	39 (16.7)				
	Bachelor’s degree	73 (31.2)				
	Graduate degree	103 (44)				
**Marital status, n (%)**						
	Married or living with partner	194 (82.9)				
	Divorced, widowed, or separated	18 (7.7)				
	Never married	19 (8.1)				
**Household annual household income (US $), n (%)**						
	<24,999	4 (1.7)				
	25,000-34,999	3 (1.3)				
	35,000-49,999	14 (6)				
	50,000-74,999	24 (10.2)				
	75,000-999,999	36 (15.3)				
	>100,000	142 (61.4)				
	Prefer not to answer	11 (4.7)				
Children’s ages (years), mean (SD)		6.5 (1.3)				
**Children’s sex, n (%)**						
	Female	133 (56.8)				
	Male	101 (43.2)				
**Children’s race and ethnicity, n (%)**						
	White	196 (83.8)				
	Black	10 (4.3)				
	Asian	1 (0.4)				
	Multiracial	24 (10.3)				
	Hispanic or Latino	11 (4.7)				

^a^Two participants from the analytic sample (N=236) did not complete the demographic survey, resulting in N=234 for analysis of participant demographics.

## Discussion

### Principal Findings

Findings hypothesized to result from this study include a greater selection of promoted healthy meal items in the intervention group versus controls and corresponding improvements in the nutritional characteristics of children’s meal orders and dietary intake. The planned research will build upon the existing evidence highlighting choice architecture and repeated exposure as potentially promising intervention approaches in restaurants, including emerging evidence demonstrating potential small effects of approaches that increase the prominence of healthier children’s menu items [18,35,36] and evidence suggesting that these impacts may be magnified if incorporating optimal defaults [12] and repeated exposure [6]. If the intervention significantly impacts the meal selected for the child, findings would support the idea that these intervention approaches can facilitate the selection of healthier meals for children in restaurants. Such findings could support restaurant usage of existing loyalty programs, placemats, and signage, not just to encourage purchasing but also to promote healthy choices.

If intervention effects within the sales data have an equal or greater magnitude compared to individual-level results, findings would support choice architecture as a promising intervention approach, given that the repeated exposure component of the intervention (the frequent diner card) was only provided to those enrolled in the study. If, by contrast, the results are of the greatest magnitude during the exposure period in the study participant sample, these findings would support the frequent diner card as a meaningful addition. If hypotheses are not confirmed, and the intervention does not impact orders or intake, these findings would suggest alternative approaches are needed. This could imply the need for an approach centered on reworking children’s menus so the majority of available options are nutritious, and all meals come with healthy sides by default [12,13,37], although it is unclear whether previous successful implementation of these strategies would generalize to a broader population of restaurants.

Strengths of this study include the cluster-randomized design and data collection within naturalistic restaurant settings that includes measurement not only of children’s orders but also gold standard measurements of dietary intake via weighed plate waste. Limitations include the possibility of demand characteristics, with participants’ or restaurant staff behaviors influenced by perceptions of the researchers’ goals, and the results may not generalize beyond regional quick-service restaurant chains similar to this partner restaurant chain or to populations beyond the demographics of those studied here. Demographics reflect that the sample is comprised of children aged 4 to 8 years (by design), with representation of both male and female children, but less racial and ethnic diversity, as most of the children were white. Future directions could include scaling the current approach, if successful, and testing its effects in different types of restaurants and among participants of different sociodemographic backgrounds. Additional possible extensions include further implementation and dissemination research, and consideration of other modalities of food purchasing, such as drive-throughs and delivery.

### Conclusions

As restaurants are normative eating contexts for many families, in-restaurant interventions that make selecting healthier options easier and more normative have the potential to have a meaningful public health impact. This study’s intervention brings together the limited extant research on promoting healthy choices for children in restaurants with approaches from the behavioral sciences —choice architecture and repeated exposure—which have been successful in other environments, such as laboratories and childcare centers. The present approach differs from existing restaurant interventions that are information-based, such as providing nutrition information via calorie labeling (eg, [38]), and has the potential to elicit change while minimizing concerted effort required from customers, a characteristic that may be important in the context of busy, quick-service restaurants. Results from this trial can inform future health promotion efforts in restaurants by shedding light on the feasibility and effectiveness of choice architecture and repeated exposure strategies in this setting.

## Appendix

Multimedia Appendix 1 Sample child interview (posttest time point, intervention group, aged 4 to 5 years).

Multimedia Appendix 2 Sample interview guide: restaurant manager.

Multimedia Appendix 3 Sample interview guide: other restaurant employees (not owner or manager).

Multimedia Appendix 4 Peer-review report by PRDP - Psychosocial Risk and Disease Prevention Study Section, Risk, Prevention and Health Behavior Integrated Review Group (National Institutes of Health, USA).

## Abbreviations

ASA24: Automated Self-administered 24-hour Dietary Assessment Tool

## Data Availability

Data are available upon reasonable request to the corresponding author after planned analyses are completed by the study team.
