# Supplementary material for: Promoting Healthier Meal Selection and Intake Among Children in Restaurants: Protocol for a Cluster-Randomized Trial
Source: JMIR Res Protoc. 2025 Oct 10;14:e73618. doi: 10.2196/73618 (PMC12552822; doi:10.2196/73618)
Supplement: Multimedia Appendix 1 [file resprot_v14i1e73618_app1.docx]

**APPENDIX 1. SAMPLE CHILD INTERVIEW (Post-test time point; intervention group; 4-to-5 year old)**

*Questions are administered verbally by study staff to the child. The study staff enters the participant ID and age of child on the electronic tablet (or on the paper survey) and then administers assent before asking the questions that follow.*

**Assent (Staff to Child): Hi, [Child Name], my name is [Name]. Can you tell me how old you are? OK, great.**

**How old is the child: _________________ years** *(confirming age for use of age appropriate assent procedure and visual face scale)*

**[Name of Lead] is talking to your [Mom/Dad/name of other guardian] about your time in Anderson's today, and I would like to hear what you think too. I have a few questions here that I will ask you about your meal here today.**

**You don't have to do anything you don't want to do, and you can let me know if you no longer want to answer questions. Would you like to go ahead and answer the questions?**

***If yes* ... Ok great (start questions on tablet)**

***If no.*.. Are you sure you don't want to answer questions like your [Mom/Dad/name of other guardian did]?**

***If still no* ... Ok, that's alright. We're going to talk to your [Mom/Dad/other guardian] for a couple more minutes, then, and your family can be on their way.**

*******

**We are here in the restaurant today to learn about kids’ meal choices. We are going to ask you some questions about the kids’ meals at this restaurant and what you ate today.**

**There are no right or wrong answers. This is not a test.**

**Please tell us what you really think. Telling us what you really think will help us understand what kids think about kids’ meals and can help restaurants to sell food that kids like you would like to get.**

**We are going to start by talking about what you had to eat in the restaurant today.**

**What did you have to eat today? [*We will not record this as we are asking the parents this too. We are asking it here to get the conversation started*].**

**Did you have anything else besides [what they just said they had]?**

**Ok.**

1. **Did you know you were going to have [restate what they had] before you got here?**
   - **Yes**
   - **No**
2. **Have you been to this restaurant before?**
   - No [Skip to 4]
   - Yes [Go to 3]
   - I don’t know [Skip to 4]
3. **Is [restate what they had] what you usually have to eat when you come to this restaurant?**
   - No [Go to 3a]
   - Yes [Go to 4]
   - I don’t know [Go to 4]

**3a.** **What do you usually have to eat when you come to this restaurant?** [open]

1. **Who picked the meal that you ordered today?**
   - I picked my meal
   - A grown-up (like Mom or Dad) picked my meal
   - A grown-up and I picked my meal together
   - Other (please specify): ___________________________

***If child is 4 or 5 years old (similar 5-point scale used for older children):***

Now I’m going to show you some faces.


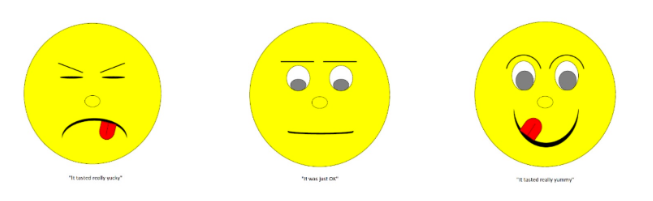


This face (point when talking about a specific face) is a really sad face that shows when you really don't like something.

This face isn't happy or sad. This face shows when you aren't sure if you like something or don't like something or if you think it is just OK.

And last, this face is really happy. This face shows when you really like something.

Now I'm going to ask you some questions about your meal and I want you to show me which face shows how you feel about each question.

1. **How did your meal taste today? [point to corresponding faces] Was it really yucky, just OK, or really yummy?**

- Really yucky
- Just OK
- Really yummy

**5a.What part of your meal tasted the yummiest? Which food/which drink? [Indicate the big smiley face.]**

**__________________________________________________________________________________**

**5b.What part of your meal tasted the yuckiest? Which food/which drink? [Indicate the big frowny face.]**

**__________________________________________________________________________________**

1. **Have you ever gotten a placemat like this here in this restaurant?**


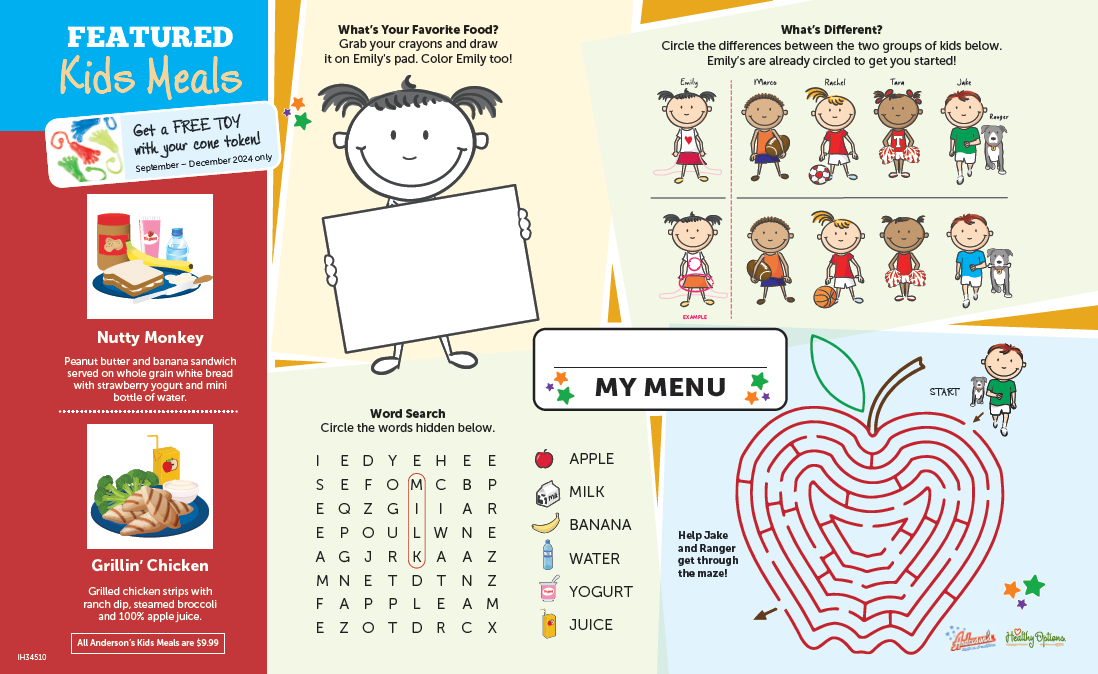


- - No [go to 8]
  - Yes
  - I don’t know [go to 8]

1. **Can you show me which face shows how much you like or don’t like the placemat? [point to corresponding faces] Do you really not like it, do you think it is just OK, or do you really like it?**


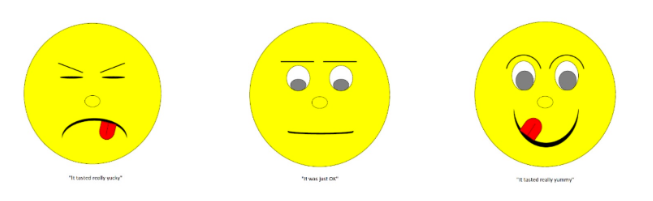


- - I really don’t like it
  - It is just ok
  - I really like it

1. **Have you ever gotten a sticker like this [show examples of study stickers] when you ordered your food?**
   - No [go to 10]
   - Yes
   - I don’t know [go to 10]
2. **Can you show me which face shows how much you like or don’t like the stickers you got when you ordered your food? [point to corresponding faces] Do you really not like it, do you think it is just OK, or do you really like it?**


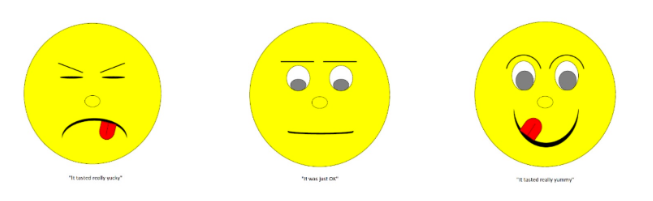


- - I really don’t like it
  - It is just ok
  - I really like it

1. **Have you ever gotten a dessert token like this [show token] with your meal?**
   - No [ask 11 only then end survey]
   - Yes [ask 11 and then 12]
   - I don’t know [ask 11 only then end survey]
2. **Did you know you could trade in your dessert token for a toy?**
   - No
   - Yes
   - I don’t know
3. **Have you ever traded in your dessert token for a toy?**
   - No [end survey]
   - Yes
   - I don’t know [end survey]
4. **What kind of toy did you get?** (select all that apply)

- Lego dinosaur
- Squishy animal (penguin/hippo)
- Sticky feet
- I don’t know

1. **Can you show me which face shows how much you like or don’t like the toy(s) you got when you traded in your dessert token? [point to corresponding faces] Do you really not like it, do you think it is just ok, or do you really like it?**


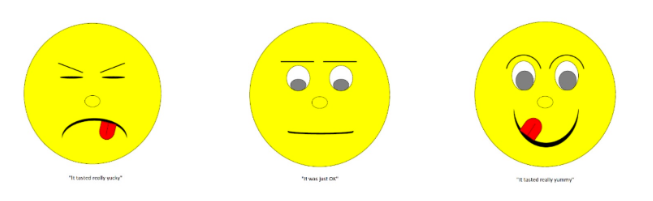


- - I really don’t like it
  - It is just ok
  - I really like it

**[End of survey]**

**OK, we are done! Thank you for helping us with our study. It was fun hearing about what you thought about your visit to the restaurant today.**
