# Supplementary material for: Promoting Healthier Meal Selection and Intake Among Children in Restaurants: Protocol for a Cluster-Randomized Trial
Source: JMIR Res Protoc. 2025 Oct 10;14:e73618. doi: 10.2196/73618 (PMC12552822; doi:10.2196/73618)
Supplement: Multimedia Appendix 2 [file resprot_v14i1e73618_app2.docx]

**APPENDIX 2: SAMPLE INTERVIEW GUIDE: RESTAURANT MANAGER**

Hello, may I please speak with [FIRST AND LAST NAME]? My name is [YOUR NAME] and I’m calling from the Restaurant Study at the University at Buffalo. You told us that this would be a good time to call you to complete your Restaurant Study interview. Will this still work for you? [*Note to Interviewer:  If participant says that they would like to reschedule, exit the survey and schedule another call with the participant at a time that will work better for them. Also make sure to introduce anyone else on the call (for example if there is a notetaker)*]

Thank you for agreeing to participate in this interview. Researchers at the University at Buffalo are talking to representatives from Anderson’s that are part of the Restaurant Study. Did you receive the information sheet we sent?

If yes: do you have any questions?

## **If no, review the following points:**

- Our goal is to better understand the factors that help or hurt the implementation of the Restaurant Study
- Interview answers will be used to improve training and delivery of the UB Restaurant Study
- Interviews will take place toward the end of the UB Restaurant Study via Zoom or telephone
- Any individual who is currently or previously employed at an Anderson’s location that is involved with the Restaurant Study this year can participate
- Participation is voluntary. Refusal to participate will not result in penalty or loss of benefits as an employee of Anderson’s
- Participants can stop the interview at any time
- Participants who complete the interview will receive $25

Do you have any questions?

Let me give you some more details about the interview process:

- Interviews will include questions about how well the study is working, how easy or hard it is to implement, and whether it is a good fit for Anderson’s
- Today’s interview should last no more than 30 minutes.
- We will be taking notes, but we want to make sure that we get everything that you say right. Are you okay with us audio-recording this interview?
- Before we start, I want you to know that there are no *right* or *wrong* answers. You are the expert here, so please share any information that you think might be helpful. It is also OK if you don’t know the answer to a question. If you want to tell me something, but you do not want it recorded, please let me know and I will stop the recording

Your agreement to participate in the interview indicates your consent to participate in this research. Would you like to take some time to think about this before beginning the interview, or are you ready to start?

*Let’s start by having you tell me a little bit about your position with Anderson’s.*

1. At which Anderson’s location do you work?
2. How long have you been working for Anderson’s
3. What roles have you had within the company during this time?

*The next questions are about the UB Restaurant Study that has been happening at your location this year.*

1. Why is the Restaurant Study being done?
   1. Probe: How was the decision made for Anderson’s to participate in the Restaurant Study?
2. What is the goal of the Restaurant Study?
   1. Do you think Anderson’s is a good place to do this study? Probe: Why/why not?
3. Who developed the Restaurant Study?
   1. What is your opinion of this group?
   2. What does Anderson’s owner think about the restaurant study?
4. On a scale of one to ten with one being very easy and ten being very hard, how easy or hard was it to complete the tasks needed for the UB Restaurant Study?
   1. What parts were easy?
   2. What parts were hard?
5. Did you have any issues keeping the menu items related to the study in stock? (For example, the strawberry yogurt tubes or the kids’ the peanut butter and banana sandwich or grilled chicken meals?)
   1. Was there demand for these items from customers?
6. After the study is over, do you think that Anderson’s should continue to do any of the things that were done for the study, such as the kids’ placemats, digital frequent diner cards that customers bring in to be scanned, **or toys** *[toys mentioned for intervention locations only]*? Why or why not?
   1. Is there anything you would change about the materials that were provided as part of the study?
   2. What should not be changed?
   3. How easy or hard do you think it would be for Anderson’s to continue doing these things after the study ends?
7. Who will decide (or what is the process for deciding) whether changes are needed in order for materials like placemats, digital frequent diner cards, **and toys** to work well at your restaurant?
   1. How will you know if it is appropriate to make any changes?
8. What is your perception of the quality of the materials that were used for the Restaurant Study?
   1. Probe: what did you think about the placemats? Digital frequent diner cards that customers brought in to be scanned? Toys? Other signage?
9. How did your employees learn about the study?
   1. What type of training did they receive about the study?
10. How much training did you get on tasks related to the study?
    1. Was it enough?
    2. Were there things you wished you would have known?
11. Who do you ask if you have questions about the UB Restaurant Study?
    1. How available are these individuals?
12. Do you think the study had any benefits for the restaurant?
    1. Probe: customer repeat visits? Revenue?

*The next questions are about how you think customers were affected by having the study at Anderson’s*

1. How much do you think that customers noticed the changes in place during the study?
   1. Did you hear any specific comments about the items related to the study, such as specific kids’ meals or yogurt tubes, **toys,** placemats, or other study materials like signage?
   2. How well do you think these items met the needs of your customers?
2. Do you think having these options was welcomed by customers? Why or why not?
3. Has your staff shared any customer feedback with you?

*The next questions are about whether or not the restaurant study is a good fit for Anderson’s right now.*

1. How well does a healthy kids’ menu program fit with the values and norms of Anderson’s?
2. How well do the placemats, digital frequent diner cards, **toys** and other study materials fit in with the way you do things?
   1. Did you find any problems with fitting the study materials and tasks into your normal activities at work? – such as the normal ordering and payment process?
3. Were there any other new initiatives happening at Anderson’s at the time of the UB Restaurant Study?
4. How important was the UB Restaurant Study compared to other new initiatives?
   1. Describe activities or initiatives that (appear to) be the highest priority for Anderson’s right now.
5. How motivated were you or your staff to ensure that the Restaurant Study was successful?
   1. What was your motivation for wanting to help ensure it was successful?
   2. Are there any special recognitions or rewards related to implementing the study?

*Thank you so much for your time to complete this interview. Your answers will help make this program better for other restaurant locations.* [Stop recording] *You will receive payment for today’s interview in the mail in about a week. Your payment will be loaded onto a US Bank Card which can be used just like a credit card or debit card by creating a pin. What address would you like this card sent to?* [also collect date of birth to set up card]
