# Supplementary material for: Promoting Healthier Meal Selection and Intake Among Children in Restaurants: Protocol for a Cluster-Randomized Trial
Source: JMIR Res Protoc. 2025 Oct 10;14:e73618. doi: 10.2196/73618 (PMC12552822; doi:10.2196/73618)
Supplement: Multimedia Appendix 3 [file resprot_v14i1e73618_app3.docx]

**APPENDIX 3: SAMPLE INTERVIEW GUIDE: OTHER RESTAURANT EMPLOYEES (NOT OWNER OR MANAGER)**

Hello, may I please speak with [FIRST AND LAST NAME]? My name is [YOUR NAME] and I’m calling from the Restaurant Study at the University at Buffalo. You told us that this would be a good time to call you to complete your Restaurant Study Interview. Will this still work for you? [*Note to Interviewer:  If participant says that they would like to reschedule, exit the survey and schedule another call with the participant at a time that will work better for them. Also make sure to introduce anyone else on the call (for example if there is a notetaker)*]

Thank you for agreeing to participate in this interview. Researchers at the University at Buffalo are talking to representatives from Anderson’s that are part of the Restaurant study. Did you receive the information sheet we sent? If yes, do you have any questions?

## **If no, review the following points:**

- Our goal is to better understand the factors that help or hurt the implementation of the Restaurant Study
- Interview answers will be used to improve training and delivery of the UB Restaurant Study
- Interviews will take place toward the end of the UB restaurant study via phone or Zoom
- Any individual who is currently or previously employed at an Anderson’s location that is involved with the Restaurant Study this year can participate
- Participation is voluntary. Refusal to participate will not result in penalty or loss of benefits as an employee of Anderson’s
- Participants can stop the interview at any time
- Participants who complete the interview will receive $25

Do you have any questions?

Let me give you some more details about the interview process:

- Today’s interview will include questions about how well the study is working, how easy or hard it is to implement, and whether it is a good fit for Anderson’s
- The interview should last no more than 30 minutes
- We will be taking notes, but we want to make sure that we get everything that you say right. Are you okay with us audio-recording this interview?
- Before we start, I want you to know that there are no *right* or *wrong* answers. You are the expert here, so please share any information that you think might be helpful. If you don’t know the answer to question, that is OK. If you want to tell me something, but you do not want it recorded, please let me know and I will stop the recording.

Your agreement to participate in the interview indicates your consent to participate in this research. Would you like to take some time to think about this before beginning the interview, or are you ready to start?

*Let’s start by having you tell me a little bit about your position with Anderson’s.*

1. At which location of Anderson’s do you currently work?
2. How long have you been working for Anderson’s?
3. What roles have you had within the company during this time?

*The next questions are about the UB Restaurant Study that has been happening at your location this year.*

1. What is the goal of the Restaurant Study?
   1. Do you think Anderson’s is a good place to do this study? Probe: Why/why not?
2. Who developed the Restaurant Study?
   1. What is your opinion of this group?
   2. What do the managers at Anderson’s think about the Restaurant Study?
3. What kinds of things were you asked to do as the study has been going on?
4. On a scale of one to ten with one being very easy and ten being very hard, how easy or hard was it to complete the tasks needed for the Restaurant Study?
   1. What parts were easy?
   2. What parts were hard?
5. Did customers ever ask for items related to the study, such as specific kids’ meals, yogurt tubes, or **toys** *[toys mentioned for intervention locations only]* but you did not have them available?
6. After the study is over, do you think that Anderson’s should continue to do any of the things that were done for the study, such as the kids’ placemats, digital frequent diner cards that customers bring in to be scanned, **or toys**? Why or why not?
   1. Is there anything you would change about the materials that were provided as part of the study?
   2. What should not be changed?
   3. How easy or hard do you think it would be for Anderson’s to continue doing these things after the study ends?
7. What is your perception of the quality of the materials that were used for the Restaurant Study?
   1. Probe: what did you think about the placemats? Digital frequent diner cards that customers brought in to be scanned? **Toys?** Other signage?
8. How much training did you get on tasks related to the study?
   1. Was it enough?
   2. Were there things you wished you would have known?
9. Who do you ask if you have questions about the UB Restaurant Study?
   1. How available are these individuals?
10. Do you think the study had any benefits for the restaurant?
    1. Probe: repeat customer visits, revenue

*The next questions are about how you think customers were affected by having the study at Anderson’s.*

1. How much do you think that customers noticed the changes in place during the study?
   1. Did you hear any specific comments about the items related to the study, such as specific kids’ meals or yogurt tubes, **toys,** placemats, or other study materials like signage?
   2. How well do you think these items met the needs of your customers?
2. How often have you provided a placemat and crayons with kids’ meals this Fall?
3. Do you recall the meals that were highlighted on the placemats?
   1. Their names? What was included?

*Intervention locations only:*

1. How often did you encourage families to choose one of the featured meals?
2. Do you recall what families had to do to get a scan of their digital frequent diner card?

*Intervention locations only:*

1. How often did you tell children/parents with tokens that they could get a toy instead of a dessert if they wished?
2. Do you think having these options was welcomed by customers? Why or why not?
3. Have you heard any customer feedback?

*The next questions are about whether or not the restaurant study is a good for Anderson’s right now.*

1. How well do the placemats, digital frequent diner cards, and other study materials fit in with the way you do things?
   1. Did you find any problems with fitting them into your normal work activities – like the normal ordering and payment process?
2. Were there any other new initiatives happening at Anderson’s at the time of the UB Restaurant Study?
3. How important was the UB Restaurant Study compared to other new initiatives?
   1. Describe activities or initiatives that (appear to) be highest priority for Anderson’s right now.
4. How motivated were you to ensure that the UB Restaurant Study was successful?
   1. What was your motivation for wanting to help ensure the study was successful?

*Thank you so much for your time to complete this interview. Your answers will help make this program better for other restaurant locations.* [stop recording] *You will receive payment for today’s interview in the mail within a week. Your payment will be loaded onto a US Bank Card which can be used just like a credit card or debit card by creating a pin. What address would you like this card sent to?* [also collect date of birth to set up the card]
