# Supplementary material for: Promoting Healthier Meal Selection and Intake Among Children in Restaurants: Protocol for a Cluster-Randomized Trial
Source: JMIR Res Protoc. 2025 Oct 10;14:e73618. doi: 10.2196/73618 (PMC12552822; doi:10.2196/73618)
Supplement: Multimedia Appendix 4 [file resprot_v14i1e73618_app4.pdf]

SUMMARY STATEMENT

PROGRAM CONTACT: (Privileged Communication) *Release Date:* 11/06/2018

Revised Date:

Application Number: 1 R01 HD096748-01A1 Principal Investigator

ANZMAN-FRASCA, STEPHANIE

Applicant Organization: STATE UNIVERSITY OF NEW YORK AT BUFFALO

Review Group: PRDP
Psychosocial Risk and Disease Prevention Study Section

Meeting Date: 10/29/2018 *RFA/PA:* PA18-480
Council: JAN 2019 *PCC:* PGNB -LE
Requested Start: 06/01/2019 *Dual PCC:* RAJ DUAL
Dual IC(s): DK
Project Title: Promoting healthier meal selection and intake among children in restaurants

SRG Action: Impact Score:19 Percentile:4
Next Steps: Visit https://grants.nih.gov/grants/next_steps.htm
Human Subjects: 30-Human subjects involved - Certified, no SRG concerns
Animal Subjects: 10-No live vertebrate animals involved for competing appl.
Gender: 1A-Both genders, scientifically acceptable
Minority: 1A-Minorities and non-minorities, scientifically acceptable
Children: 2A-Only Children, scientifically acceptable

Project Year	Direct Costs	Estimated Requested	Total Cost
1			
2			
3			
4			
5			
TOTAL			

ADMINISTRATIVE BUDGET NOTE: The budget shown is the requested budget and has not been adjusted to reflect any recommendations made by reviewers. If an award is planned, the costs will be calculated by Institute grants management staff based on the recommendations outlined below in the COMMITTEE BUDGET RECOMMENDATIONS section.

EARLY STAGE INVESTIGATOR
NEW INVESTIGATOR

1R01HD096748-01A1 Anzman-Frasca, Stephanie

EARLY STAGE INVESTIGATOR
NEW INVESTIGATOR

RESUME AND SUMMARY OF DISCUSSION: This application proposes to conduct a cluster randomized clinical trial that tests a choice architecture intervention featuring placemats depicting healthy meal options to influence the meal choices of 4 to 8 year old children and tests a frequent diner

card program. This resubmitted work was highly responsive to previous critiques resulting in work that the review panel judges to offer high impact in its significant focus on the public health challenge of childhood obesity. Reviewers again noted many strengths in this well written application: the outstanding significance and scientific premise of testing healthier meal selection using choice architecture to promote early childhood healthier eating in one of the first tests of a restaurant setting; the exceptional team of investigators and research environment; and the outstanding innovation inherent in the appeal of healthy choice placemats for children and use of punch cards to promote repeated exposure. The scientific rigor of the approach is also outstanding, strengthened by the cluster RCT study design, follow up assessment to assess child choice and dietary intake, well matched control condition, strong pilot data and formative work, strong partnership with the restaurant chain, readily disseminable design if effective, excellent retention plan, and objective sales data and plate waste assessment. Some minor weaknesses discussed were questions about why conduct two rather than a single 24 hour recall, under detailed assessment of children’s willingness to engage, and the role of the punch card in motivating the child if the restaurant choice is the parents not the childs. In sum, the reviewers termed this high impact work interesting and exciting with high potential to impact the prevention of childhood obesity.

DESCRIPTION (provided by applicant): US children’s diets are unhealthy, a critical issue to address in the context of the obesity epidemic. Restaurants are one setting in which environmental shifts could promote healthier eating among children. One-third of children eat food from quick-service (fast food) restaurants on a given day, and consumption of restaurant food has been linked with increased energy intake and poorer diet quality. Taste is a key factor influencing food choices for children in restaurants, so approaches that increase children’s liking of healthier options offer promise to improve their eating behavior in these settings and more broadly. The goal of the proposed research is to make healthier options more appealing and easier to choose via an in-restaurant intervention that combines choice architecture and repeated exposure strategies. The choice architecture element will be a placemat designed to make it easier to choose healthier kids’ meal items and omit dessert. The repeated exposure element will be frequent diner cards, designed to increase exposure to and enjoyment of target foods. Extensive basic research supports repeated exposure as a powerful strategy to improve children’s taste preferences. Further, in a pilot study of 58 families in a quick-service restaurant, we showed the promise of our planned intervention: Children provided with placemats promoting healthier kids’ meals ordered a greater number of healthier foods than controls, and children who ordered promoted main dishes consumed less saturated fat than those who did not. Our long-term goal is to establish the effectiveness of an in-restaurant healthy eating intervention that can be implemented and disseminated across restaurants. The objective of this grant is to expand on our preliminary research by rigorously testing effects of an intervention that combines choice architecture and repeated exposure strategies on children’s food selection and intake in quick-service restaurants and throughout the day. We will randomize restaurant locations to groups, and children who visit restaurants regularly (n=930; 310 per cohort) will be exposed to placemats and frequent diner cards promoting healthier meals or generic (control) versions of these materials. The frequent diner cards will incentivize selection of a healthier featured meal over 6 occasions in the intervention group. The central hypothesis is that after these exposures, intervention group children will be more likely to select a healthier meal. Children’s meal selection and dietary intake in the restaurant and throughout the day will be assessed at initial and post-test time points to test hypotheses that this intervention promotes healthier meal selection (Specific Aim 1) and dietary intake (Specific Aim 2). Families’ behaviors at interim time points and potential impacts on restaurants will also be monitored. Given how frequently children eat food from restaurants, the typical consumption patterns while there, and the promise of repeated exposure to impact eating behaviors broadly, this intervention has the potential for meaningful public health impact. The present approach is novel in its application of behavioral strategies and offers the potential for lasting impacts on children’s diets and health.

PUBLIC HEALTH RELEVANCE: More than one-third of children eat food from restaurants on a given day, and consumption of restaurant food has been linked with increased energy intake and poorer diet quality. The goal of this application is to test an in-restaurant intervention that aims to use behavioral strategies, specifically choice architecture and repeated exposure, to promote healthier meal selection and dietary intake among children. This study will be the first to apply these evidence-based behavioral strategies in a randomized study within restaurants and offers the potential for a meaningful impact on children's diets and health.

CRITIQUE 1

Significance: 3
Investigator(s): 2
Innovation: 2
Approach: 2
Environment: 1

Overall Impact: This revised R01 tests an intervention designed promote purchasing of healthy kids meals in restaurants. The team was very responsive to the prior round of reviews. The likelihood of achieving a meaningful impact on diet and obesity through this intervention is unclear, but the simplicity of the intervention enhances its dissemination potential greatly. The study has strong scientific rigor, including a thorough assessment plan for secondary outcomes and aspects of program implementation. The partnership with the restaurant chain is a key asset of the project. Overall this project is poised to have high scientific impact, and moderate public health impact.

1. Significance:

Strengths

- The study leverages a valuable academic-private partnership to test a novel intervention in the real world
- This approach would have high potential for dissemination

Weaknesses

- The intervention may result in slight improvements in meals chosen when eating out, but may not have a noticeable overall impact on obesity or diet quality (minor weakness)

2. Investigator(s):

Strengths

- PI has demonstrated expertise in children’s eating patterns and associated interventions.
- Team includes experiences obesity researchers and behavioral trialists, and a statistician.

Weaknesses

None noted.

3. Innovation:

Strengths

- This is one of the first real-world intervention studies of a choice architecture program targeting kid’s restaurant meal choices.
- The ability of a child to swap out dessert for a toy is a novel idea worth testing.

Weaknesses

- Choice architecture and menu design strategies are not necessarily innovative

4. Approach:

Strengths

- The cluster-randomized design will include 6 restaurant locations
- Team will conduct taste tests of promoted items to ensure their acceptability
- Study will utilize objective sales data collected from the restaurants
- There are plans to assess plate waste, and the nutrient content of consumed meals, and children dietary intake
- Protocol includes observations of family interactions and assessment of program implementation

Weaknesses

- There is some uncertainty about whether the frequent diner program really translates into repeated food exposures within a learning and memory framework (minor weakness)
- It will be difficult to determine the extent to which participation in the program is driven by its appeal to consumers, or the study's retention protocol (minor weakness)

5. Environment:

Strengths

- University of Buffalo has a strong infrastructure for the project.
- Clear evidence of a partnership with the study site – Anderson's Frozen Custard – and 6 restaurant locations will participate.

Weaknesses

- None noted.

Study Timeline: Strengths

- Timeline is appropriate and includes all key study milestones

Weaknesses

- None noted.

Protections for Human Subjects:

Acceptable Risks and/or Adequate Protections

- Minimal risk study

Data and Safety Monitoring Plan (Applicable for Clinical Trials Only):

Acceptable ○ Monitored by investigators

Inclusion of Women, Minorities and Children:

- Sex/Gender: Distribution justified scientifically
- Race/Ethnicity: Distribution justified scientifically
- For NIH-Defined Phase III trials, Plans for valid design and analysis: Not applicable
- Inclusion/Exclusion of Children under 18: Including ages <18; justified scientifically Kids 4-8, parents. Both genders, all race/ethnicities

Vertebrate Animals:

Not Applicable (No Vertebrate Animals)

Biohazards:

Not Applicable (No Biohazards)

Resubmission:

- Very responsive to the prior round of reviews

Budget and Period of Support:

Recommend as Requested

CRITIQUE 2

Significance: 1
Investigator(s): 1
Innovation: 1
Approach: 2
Environment: 1

Overall Impact: This revised/resubmitted proposal describes a choice architecture intervention that is designed to examine the effects of adding healthy placemats and frequent diner cards on 4- to 8-yearold children's food selection and dietary intake at quick-service restaurants. Strengths include the stellar investigative team and the innovative use of choice architecture to influence children's food choices and eating patterns in restaurants. Scientific rigor is high; the application outlines the team's formative and process evaluation approaches, as well as randomization and matching procedures. The investigators responded well to the previous concerns and suggestions of the reviewers; most of the changes made have improved the quality of the proposal and the potential for success. However, one of the incorporated changes (the addition of 24-hour dietary recall), is questionable and not welljustified. In addition, the potential influence of restaurant employees on study outcomes was not adequately addressed. These and other concerns are mostly minor; enthusiasm is high for this exciting, potentially high-impact proposal.

1. Significance:

Strengths

- Eating away from home, particularly fast-food intake, is a risk for poor dietary patterns and obesity.
- Choice architecture may be a cost-effective method for increasing children's healthy meal/food selections at restaurants.

- Scientific premise is sound; little work has been conducted on the influence of repeated exposure on children's food choices in the context of a restaurant. The PI has conducted pilot work in this area, which her work in engineering choice architecture in restaurants to impact young children's meal choices/intake.
- Data from the pilot study suggest the potential for high impact.

Weaknesses

None noted

2. Investigator(s):

Strengths

- The team is stellar. Dr. Anzman-Frasca is a productive early-career investigator with an impressive record of research on in-restaurant interventions, and repeated vegetable exposure on intake in young children.
- Additional team members add considerable strength in the areas of the behavioral economics of eating behavior (Dr. Epstein), community-based research (Dr. Leone), biostatistics (Mr. Paluch) and measurement of dietary intake (Dr. Haines).
- Letters of support provide evidence of support for the project from the regional restaurant chain (Anderson's), and well as a company offering design services and dietetic consultation (Independent Health Foundation).
- The university-community partnership (with the restaurant) adds great strength.

Weaknesses

None noted.

3. Innovation:

Strengths

- This study, if successful, would make a great contribution to the literature on in-restaurant interventions that use choice architecture to influence children's meal/food selections. There is little information on this topic, apart from the published pilot study led by the PI.
- Repeated exposure in the form of a frequent diner card is novel. **Weaknesses** None noted

4. Approach:

Strengths

- Matching intervention components (both groups receiving a placemat and frequent diner card that vary based on the focus on healthy meals) is a strength.
- The data analysis plan is sound.
- A commitment from the restaurant to remove/not include any other marketing towards children is a strength.
- Adding the option for children to choose a toy over a dessert is a significant improvement over the previous version and adds strength to the study design.
- Online surveys throughout the 6-week intervention.
- Observing parent AND child ordering/eating behavior at the restaurant adds strength.

Weaknesses

- If food will be post-weighed, and intake calculated based on pre-weight estimates, it is unclear why plate waste methods to estimate intake will also be used. (minor concern – issue of unnecessary investigator burden)
- Although suggested by a previous reviewer, it is unclear how a 24-hour recall at pre- and posttest adds strength to the study. It would be good to incorporate recall-like questions to the online surveys, but unless families are assessed immediately following a visit to the restaurant, its utility is questionable. It is unclear what is meant (in the section on the ASA24) by “the first day will include the restaurant meal consumed at that time point.” (minor to moderate concern)
- It may be important to assess children’s willingness to choose a healthy meal at the restaurant during the 6 online surveys. May be interesting to understand how much coercing parents had do to, vs. children willingly choosing the healthy option. (minor concern)

5. Environment:

Strengths

- The University of Buffalo will provide excellent support for this project.
- The letter of support from Anderson’s provides evidence of a commitment of full support for the project.

Weaknesses

- None noted.

Study Timeline:

Strengths

- The study timeline is adequate to achieve the aims of this project.

Weaknesses

- None noted.

Protections for Human Subjects:

Acceptable Risks and/or Adequate Protections

- NOTE: Restaurant staff will be trained on implementation. Given that they will not be blinded to study condition, what efforts will be made to prevent them from altering study results?

Data and Safety Monitoring Plan (Applicable for Clinical Trials Only):

Acceptable ○ Internal monitoring will be conducted by the study team.

Inclusion of Women, Minorities and Children:

- Sex/Gender: Distribution justified scientifically
- Race/Ethnicity: Distribution justified scientifically

- For NIH-Defined Phase III trials, Plans for valid design and analysis: Not applicable
- Inclusion/Exclusion of Children under 18: Including ages <18; justified scientifically
- The sample will include parents (mostly mothers) of male and female children ages 4-8 years; the sample will be diverse, with an estimated 45% from minority groups.

Vertebrate Animals:

Not Applicable (No Vertebrate Animals)

Biohazards:

Not Applicable (No Biohazards)

Resubmission:

- The investigators responded extremely well to the previous concerns of the reviewers. Almost all, if not all suggestions were incorporated.

Resource Sharing Plans:

Acceptable

Budget and Period of Support:

Recommend as Requested

CRITIQUE 3

Significance: 1
Investigator(s): 1
Innovation: 2
Approach: 2
Environment: 1

Overall Impact: This is a highly responsive resubmission application of high impact from a promising early stage investigator. She is joined by an accomplished team with relevant expertise. They propose a cluster RCT of an intervention based on choice architecture and repeated exposure strategies designed to nudge 4-8 years old children towards healthier fast food choices. The intervention uses placemats featuring two healthier kids' meals and 2) frequent diner cards which reward kids for choosing healthier kid meal options after 6 exposures. Participants will already need to eat out frequently (1/week or more) to enroll. Scientific premise is very solid, and the PI contributes many peerreviewed studies as well as a short-term pilot RCT for the proposed intervention in the same fast food setting. The primary outcome is selection of the promoted healthier kids' meals. Secondary outcomes include ordering and consuming fewer calories, saturated fat, etc. Plate waste will measure the actual fast food meal intake, while 24-hour recalls will measure overall dietary intake. This cluster RCT is of a highly rigorous design and will likely be highly impactful for public health and informative to quick service restaurants.

1. Significance:

Strengths

- This RCT will inform both public health efforts to improve children's dietary intake at fast food restaurants, and also provide the fast food industry with valuable information regarding the intervention's impact on sales.

Weaknesses

- Minor: the restaurant chain involved is a regional chain and efficacy/implementation in larger chains may be substantially different

2. Investigator(s):

Strengths

- The PI has conducted multiple studies on this topic, including the pilot RCT that was recently published
- The investigative team is accomplished and has the expertise to carry out the study
- The restaurant chain participated in the pilot RCT and is an enthusiastic partner for the present larger and longer study.

Weaknesses

None noted.

3. Innovation:

Strengths

- The choice architecture and repeated exposure strategy is an innovative combination

Weaknesses

- Minor: An electronic/smartphone-based loyalty card would be more innovative

4. Approach:

Strengths

- The cluster RCT design is highly rigorous and analyses appear appropriate to the design
- The intervention has been pilot tested in the same setting as the proposed cluster RCT
- 24-hour dietary recall and plate waste methods are rigorous for field studies
- The design of the healthier meals/side dishes includes formative work with the target population
- The intervention approach is based on choice architecture and repeated exposure strategies, which have a good evidence base
- The influence of parents' meal choices on their children's meal choices will be examined

Weaknesses

- The ASA-24 has mixed results for validity when used with children

5. Environment:

Strengths

- The restaurant chain has agreed to continue its partnership with the investigative team and provides an excellent environment for this study
- SUNY Buffalo provides all needed resources for this study

Weaknesses

- None noted.

Study Timeline:

Strengths

- Appropriate timeline, ambitious recruitment plan

Weaknesses

- None noted.

Protections for Human Subjects:

Acceptable Risks and/or Adequate Protections

Data and Safety Monitoring Plan (Applicable for Clinical Trials Only):

Acceptable

Inclusion of Women, Minorities and Children:

- Sex/Gender: Distribution justified scientifically
- Race/Ethnicity: Distribution justified scientifically
- For NIH-Defined Phase III trials, Plans for valid design and analysis: Not applicable □
  Inclusion/Exclusion of Children under 18: Including ages <18; justified scientifically

Vertebrate Animals:

Not Applicable (No Vertebrate Animals)

Biohazards:

Not Applicable (No Biohazards)

Resubmission:

- This application is highly responsive

Budget and Period of Support:

Recommend as Requested

THE FOLLOWING SECTIONS WERE PREPARED BY THE SCIENTIFIC REVIEW OFFICER TO SUMMARIZE THE OUTCOME OF DISCUSSIONS OF THE REVIEW COMMITTEE, OR REVIEWERS' WRITTEN CRITIQUES, ON THE FOLLOWING ISSUES:

PROTECTION OF HUMAN SUBJECTS: ACCEPTABLE

INCLUSION OF WOMEN PLAN: ACCEPTABLE

INCLUSION OF MINORITIES PLAN: ACCEPTABLE

INCLUSION OF CHILDREN PLAN: ACCEPTABLE

COMMITTEE BUDGET RECOMMENDATIONS: The budget was recommended as requested.

Footnotes for 1 R01 HD096748-01A1; PI Name: Anzman-Frasca, Stephanie

NIH has modified its policy regarding the receipt of resubmissions (amended applications). See Guide Notice NOT-OD-14-074 at http://grants.nih.gov/grants/guide/notice-files/NOT-OD14-074.html. The impact/priority score is calculated after discussion of an application by averaging the overall scores (1-9) given by all voting reviewers on the committee and multiplying by 10. The criterion scores are submitted prior to the meeting by the individual reviewers assigned to an application, and are not discussed specifically at the review meeting or calculated into the overall impact score. Some applications also receive a percentile ranking. For details on the review process, see http://grants.nih.gov/grants/peer_review_process.htm#scoring.

MEETING ROSTER

Psychosocial Risk and Disease Prevention Study Section
Risk, Prevention and Health Behavior Integrated Review Group
CENTER FOR SCIENTIFIC REVIEW
PRDP
10/29/2018 - 10/30/2018

Notice of NIH Policy to All Applicants: Meeting rosters are provided for information purposes only. Applicant investigators and institutional officials must not communicate directly with study section members about an application before or after the review. Failure to observe this policy will create a serious breach of integrity in the peer review process, and may lead to actions outlined in NOT-OD-14-073 at https://grants.nih.gov/grants/guide/notice-files/NOT-OD-14-073.html and NOT-OD-15-106 at https://grants.nih.gov/grants/guide/notice-files/NOT-OD-15-106.html, including removal of the application from immediate review.
